# On the Laryngoscope and Its Employment in Physiology and Medicine

**Published:** 1861-03

**Authors:** 


ART. V.—*Du Laryngoscope et de son Emploi en Physiologie et en Médecine.* Par J. N. CZERMAK, M.D. 8vo., pp. 112. Paris, 1860. *On the Laryngoscope and its Employment in Physiology and Medicine.*

ONE of the distinguishing features of the present day is the aid which the diagnosis of disease has received from mechanical appliances. The speculum, although dating back to the most flourishing period of Herculaneum, may be said to be, in the true sense of the term, a modern device, as far as its application is concerned to the elucidation of the nature and treatment of the disorders of the vagina and uterus. The anal speculum was but little employed thirty years ago, and of the application of the aural speculum hardly anything was known until the publication of Dr. Kramer's treatise on the diseases of the ear. Within the last few years a valuable addition has been made to our diagnostic armamentarium by the invention of the ophthalmoscope, and to a still more recent period we are indebted for the introduction of the laryngoscope and rhinoscope, whose uses in physiology and medicine the work whose title stands at the head of this article seeks to explain and elucidate.

Our author is Professor of Physiology in the University of Pesth, and it is quite apparent that he has made himself thoroughly acquainted with the larynx in its healthy and morbid relations. The work was originally issued at Leipsic, in 1860, was favorably reported upon by the Academy of Sciences and the Academy of Medicine of Paris, and was translated, during the author's sojourn in the French metropolis, by his friend, Dr. Mendl, having received important additions in its passage through the press, based upon observations made by Dr. Czermak, since the appearance of the German edition. It is accompanied by two plates illustrative of the manner of applying the laryngoscope and of the interior of the healthy larynx, and by thirty-one figures interspersed through the text, explanatory of instruments and various lesions of the larynx.

Dr. Czermak, in tracing the history of the laryngoscope, ascribes to the late Mr. Robert Liston the first idea of the application of an instrument to the diagnosis of the diseases of the larynx. In his Practical Surgery, published in 1840, the English surgeon, speaking of ulceration of the glottis, observes:—

"A view of the parts may be sometimes obtained by means of a speculum; such a glass as is used by dentists on a long stalk, previously dipped in hot water, introduced with its reflecting surface downward, and carried well into the fauces."

In 1855, Garcia published in the London Philosophical Transactions and Journal of Science the results of a series of laryngoscopic researches upon the formation of the human voice. But these remarks and observations seemed to have attracted very little attention, and they were therefore soon forgotten. In 1857, however, Dr. Turck, Physician-in-Chief of the General Hospital of Vienna, became interested in the subject, and he accordingly examined a number of cases of diseases of the larynx with the speculum, the results of which he laid soon afterward before the Imperial Royal Medical Society of that city. The observations of Czermak were commenced in the same year, and were finally embodied in the present monograph, the most complete that has yet appeared upon the subject. In addition to these writers several others have appeared, as Semeleder, Neudoerfer, Stoerk, and Gerhardt, furnishing us with quite an extensive literature upon laryngoscopy.

The work of Czermak closes with an appendix, comprising communications from the Academy of Sciences and of the Academy of Medicine of Paris, respecting the priority of his claims over those of Dr. Turck to the practical application of the laryngoscope. From the facts therein detailed it may justly be assumed that our author is fully entitled to the entire credit of this matter, it being clearly proved that all the earlier publications of his Vienna rival date subsequent to his own.

The instruments necessary for making an examination of the parts constituting the larynx and trachea, are a speculum and a mirror, by means of which the structures of the pharynx and larynx are illuminated. The speculum consists of nothing more than a square polished steel mirror with rounded edges, the most suitable diameter of which is eight lines, by one line in thickness. It may, however, vary in diameter from five to fifteen lines. It has attached to one of its angles a rod about five inches long, and so flexible that it will admit of any curve. This rod is fitted to a handle and fastened by means of a screw. To prevent the mirror becoming dim by condensation of the expired air on its face, it should be heated to as high a degree as can be borne by the patient, by plunging it into hot water, or by holding its polished surface over the flame of a spirit-lamp.

The illuminating or reflecting mirror consists of a slightly concave glass, which in the instrument before us is three inches and a quarter in diameter, with a central perforation one quarter of an inch wide. It is fitted in a light metallic frame, which is freely movable upon a rod with two lateral branches. The rod is secured in a mouth piece, which is to be held between the molar teeth. M. Czermak prefers this form of mirror, although we may employ the frontal band of Kramer, consisting of a padded strap to pass around the head, and a cushion in front, which is to be placed over the eye of the observer, and to which the same form of mirror is attached. The light of the sun may be used as the source of illumination; but as it is not always possible to obtain this, we may substitute for it the flame of an argand lamp, or of gas, thrown upon the larynx speculum, and the illumination may be rendered more intense by concentrating the flame upon the concave reflecting mirror by means of a large bi-convex lens.

In making the examination, the lamp should be placed on a table to the right of, and a little behind the patient, so that the flame may be on a level with the roof of the mouth. The patient being seated on a chair, in front of the observer, places his hands upon his own knees, inclines the body forward, slightly extends the head, so as to make the neck more prominent, and, holding his mouth widely open, flattens his tongue, at the same time drawing it a little backward. The observer places his left hand on the shoulder of the person experimented upon, and fixes his neck and chin; or this hand may be used to control the tongue with a depressor. With the right hand he introduces the larynx speculum, holding the reflecting mirror between his teeth, and looking through the central perforation. The light and the position of the patient and observer having been properly regulated, the latter heats the speculum to the proper temperature, which he knows by touching it with his finger, and requests the patient alternately to take a deep inspiration and to pronounce the vowels *a*, *e*. At this time he endeavors to place the back of the instrument against the velum and uvula, raising these structures slightly and at the same time giving the proper inclination to the speculum, being careful not to touch the posterior wall of the pharynx. The speculum is then illuminated by the rays of light reflected from the large mirror, and throwing incident luminous rays upon the larynx, reflects the image of the parts into the eye of the observer.

As the structures of the larynx are seen upside down in the reflected image, beginners are advised to study laryngoscopy upon the cadaver or upon the excised larynx. When it is impossible to obtain the human subject, Dr. Stoerk recommends physicians to experiment upon the larynx of dogs, pigs, or sheep; but the author prefers the examination to be made upon the person of the observer. The whole apparatus is extremely simple, and with a little practice any one can soon learn to apply it.

Dr. Czermak also discusses rhinoscopy, or the examination of the nasal cavities with the speculum. As the subject, however, has not been elaborated, we content ourselves with simply mentioning the fact, hoping that some one else may soon direct special attention to it.

In conclusion, we trust that the little monograph of Dr. Czermak may be reproduced in this country in an English dress, satisfied that, from the interest and novelty of the subjects discussed in it, it would meet with many readers.

